# UP601, a standardized botanical composition composed of *Morus alba*, *Yerba mate* and *Magnolia officinalis* for weight loss

**DOI:** 10.1186/s12906-017-1627-1

**Published:** 2017-02-16

**Authors:** Mesfin Yimam, Ping Jiao, Mei Hong, Lidia Brownell, Young-Chul Lee, Eu-Jin Hyun, Hyun-Jin Kim, Jeong-Bum Nam, Mi-Ran Kim, Qi Jia

**Affiliations:** 1 Unigen, Inc., 3005 1st Avenue, Seattle, WA 98121 USA; 2 Unigen, Inc., #450-86, Maebong-Ro, Dongnam-Gu, Cheonan-Si, Chungnam 330-863 Korea

**Keywords:** High-fat high-fructose-induced obesity, Obesity, Plant extract, Mouse

## Abstract

**Background:**

The prevalence of obesity is surging in an alarming rate all over the world. Pharmaceutical drugs are considered potential adjunctive therapy to lifestyle modification. However, for most, besides being too expensive, their long term usages are hindered by their severe adverse effects. Here we describe the effect of UP601, a standardized blend of extracts from *Morus alba, Yerba mate and Magnolia officinalis,* in modulating a number of obesity-related phenotypic and biochemical markers in a high-fat high-fructose (HFF)-induced C57BL/6J mouse model of obesity.

**Method:**

Adipogenesis activity of the composition was assessed in 3T3-L1 cells in vitro. Effects of UP601 on body weight and metabolic markers were evaluated. It was administered at oral doses of 300 mg/kg, 450 mg/kg and 600 mg/kg for 7 weeks. Orlistat (40 mg/kg/day) was used as a positive control. Body compositions of mice were assessed using dual energy X-ray absorptiometry (DEXA). Serum biomarkers were measured for liver function and lipid profiling. Relative organ weights were determined. Histopathological analysis was performed for non-alcoholic steatohepatitis (NASH) scoring.

**Results:**

UP601 at 250 μg/ml resulted in 1.8-fold increase in lipolysis. Statistically significant changes in body weight (decreased by 9.1, 19.6 and 25.6% compared to the HFF group at week-7) were observed for mice treated with UP601 at 300, 450 and 600 mg/kg, respectively. Reductions of 9.1, 16.9, and 18.6% in total cholesterol; 45.0, 55.0, 63.6% in triglyceride; 34.8, 37.1 and 41.6% in LDL; 3.2, 21.6 (*P* = 0.03) and 33.7% (*P* = 0.005) in serum glucose were observed for UP601 at 300, 450 and 600 mg/kg, respectively. Body fat distribution was found reduced by 31.6 and 17.2% for the 450 mg/kg UP601 and orlistat, respectively, from the DEXA scan analysis. Up to an 89.1% reduction in mesenteric fat deposit was observed for UP601 in relative organ weight. Statistically significant improvements in NASH scores were observed for mice treated with UP601.

**Conclusion:**

UP601, a standardized botanical composition from *Morus alba*, *Yerba mate* and *Magnolia officinalis* could potentially be used for achieving healthy weight loss and maintenance.

## Background

According to recent epidemiologic reports, the prevalence of obesity is surging at an alarming rate all over the world. For example, a recent study pooling data from 186 countries from 1975–2014, reported that the global obesity rates increased from 3.2% in 1975 to 10.8% in 2014 in men and from 6.4% in 1975 to 14.9% in 2014 in women [1]. Unless the current trends are reversed, it will incur significant impact on global health and the economy. It is mainly caused by an imbalance between energy intake and expenditure complicated by a sedentary lifestyle in association with easy access to a highly palatable, energy-dense diet rich in fat, sugar and salt. Untreated it can result in dyslipidemia, cardiovascular disease (including hypertension, stroke, and myocardial infarction), insulin resistance, impaired glucose metabolism, osteoarthritis and some cancers [2].

While maintaining an optimum bodyweight may seem to be a sound solution to counteract obesity, for most individuals, achieving lifestyle modifications becomes an uphill battle once they are obese. At this point, pharmaceutical drugs are considered potential adjunctive treatment to lifestyle modification. However, the lack of sustainable weight loss, cost and adverse effects render current pharmacotherapy weight management unsuccessful. Therefore, in light of the limited pharmaceutical drug choices and the socio-economic implications of the obesity pandemic, the search for safe and effective alternatives from natural sources has gained appeal within the dietary supplement industry. In this regard, we postulated that combining plant materials with similar traditional usage and safety data would give a beneficial boost in their indication for treatment of metabolic disorders. To test this hypothesis, we recently screened a series of plant extracts collected through legacy mining in vivo for their metabolic disorder related activities using a high-fat diet (HFD)-induced mouse obesity model which led to the discovery of a composition designated as UP601. UP601 is composed of standardized extracts from *Morus alba*, *Ilex paraguariensis* and *Magnolia officinalis.* These botanicals are particularly attractive because they have a long history of safe consumption, and also may provide other added health benefits beyond weight control. Previously we have reported the appetite suppression and anti-obesity effects of this composition in acute feed intake rat model and high-fat diet (HFD)-induced obese C57BL/6J mouse model [3].

*Morus alba* L (Family: Moraceae), the mulberry or white berry plant, is native to northern China, and has been cultivated and naturalized elsewhere, from India through the middle east to Southern Europe, and recently to North American. The root-bark of *Morus alba* that is used in traditional medicine is known as Sang Bai Pi or Cortex Mori (Pharmacopoeia of the People’s Republic of China, 2005). This herb is also known as Pong-na-moo in Korean and Sohakuhi in Japan. In contemporary pharmacological research, *Morus alba* root-bark has been reported to have antibacterial [4], antioxidant and hypoglycemic [5, 6] hypolipidemic, neuroprotective, antiulcer, analgesic [7–9] and anti-inflammatory activities [10]. Some of the prenylated flavonoids and stilbenoids such as morusin and mulberroside A are unique to Morus plants [11].

*Yerba mate (Family: Aquifoliaceae, botanical name: Ilex paraguariensis A. St.-Hil)* is a widely-cultivated, medium-sized evergreen tree indigenous to Paraguay, Brazil, Argentina, and Uruguay; however, it is now cultivated in many tropical countries to supply a world demand for its leaves. As a folk medicine, it has been used to suppress appetite, stimulate digestion, and as an antioxidant. Its chemical constituents were isolated and identified as xanthines, polyphenols, caffeoyl derivatives and saponins [12]. It has been reported to have antioxidant [13], lipid lowering [14], anti-cancer [15] and anti-diabetic properties [16].

*Magnolia officinalis* Rehder & E.H. Wilson (Family: Magnoliaceae) has long been traditionally used as a Chinese medicinal herb for the treatment of fever, headache, anxiety, diarrhea, stroke, and asthma. Honokiol and magnolol are considered as the two major bioactive constituents [17] and have been reported with various biological effects such as anti-inflammatory and analgesic [18], smooth muscle relaxant and antithrombotic [19, 20] treatment of diabetes and diabetic complications [21], inhibition of the formation of advanced glycation end products (AGEs) [22], antidepressant [23], anti-cancer [24] and as antioxidants [25].

It has been reported that the co-administration of high-fat and high-carbohydrate diet in animals leads to the development of typical metabolic complications present in human metabolic syndrome including obesity, hyperglycemia, dyslipidemia (exhibited as hypertriglyceridemia, hypercholesterolemia), and fatty liver [26, 27]. Hence, an obesity model induced by feeding the combination of high-fat with high-fructose instead of the high-fat or high-fructose diet alone may provide a better tool to understand the mechanisms involved in disease initiation and progression as it simulates the current calorie-rich diet of the western world. To the best of our knowledge, until recently, the disclosed plant materials have never been formulated together for weight management indications. The present study was therefore designed to assess the effect of a specific blend of these extracts on modulating a number of obesity-related phenotypic and biochemical markers in a high-fat and high-fructose (HFF)-induced C57BL/6J mouse model of obesity.

## Methods

### Material preparation

Detailed procedures of composition matter preparations have been described on US patent application entitled “COMPOSITIONS AND METHODS FOR MANAGING WEIGHT” with publication number: 20140004215.

Dried bark of *Magnolia officinalis* and dried root bark of *Morus alba* were collected from Chongqing, China and identified by professor Shou-Yuen Zhao from Si-Chuan Chinese Traditional Medicine Research Institutes. A voucher of specimen of *Magnolia officinalis* (P00491) and *Morus alba* (P00329) were deposited at the plant library of Unigen, Seattle, WA, USA. When re-collected, *Magnolia officinalis* barks and *Morus alba* root barks were always characterized and confirmed in comparison with the original voucher specimens. Dried *Ilex paraguayensis* leaves extracts were obtained from Naturex (NJ, USA).

UP601 is a proprietary blend of standardized extracts from *Magnolia officinalis* stem bark, *Morus alba* root bark, and *Ilex paraguariensis* leaf with not less than 7% magnolol and honokiol, 2% caffeine, and 1% total bioflavonoids including kuwanon G and albanin G and morusin.

*Morus alba* root bark extract was produced by 70% ethanol extraction of the ground root bark powder with no less than 10% total bioflavonoids including kuwanon G, albanin G, and morusin. *Magnolia officinalis* stem bark was extracted by a supercritical fluid and further crystalized to give a mixture of magnolol and honokiol with content higher than 95%. *Ilex paraguayensis* leaf was extracted with water to give Yerba Mate extract containing not less than 4% caffeine. The dried powders of *Magnolia officinalis* stem bark extract, *Morus Alba* root bark extract, and *Ilex paraguariensis* leaves extracts were mixed at a proprietary ratio to produce the standardized UP601 composition.

### Effects of UP601 in 3T3-L1 cells

3T3-L1 murine embryo fibroblast cells were purchased from American Type Culture Collection (Rockville, MD). Cells were cultured in Dulbecco’s modified Eagle’s medium (DMEM) (GIBCO) containing 10% bovine calf serum until confluent. Two days after post confluence (D0), the cells were stimulated to differentiate with DMEM containing 10% fetal bovine serum (FBS), 5 μg/ml insulin, 0.5 mM 3-isobutyl-1-methylxanthine (IBMX) and 1 μM dexamethasone for two days (D2). Cells were then maintained in 10% FBS/DMEM medium with 5 μg/ml insulin for another two days (D4), followed by culturing with 10% FBS/DMEM medium for four days (D8). To determine the extent of lipolysis induced by UP601, fully differentiated adipocytes (mature adipocytes) were treated with UP601, at 250 μg/ml, for 24 h and 48 h with serum free DMEM. The conditioned medium was removed from each well and free glycerol was assayed by using a Lipolysis assay kit (#F6428, Sigma-Aldrich Inc., USA) following the manufacturer’s instructions.

### Induction and intervention

The study was designed and executed in a high-fat and high-fructose (HFF) diet-induced obesity model to simulate the western pattern diet [28]. Mice were provided with 60% high-fat diet (Research diet D12492, Doo Yeol Biotech) and 30% fructose (ADM CORNSWEET®, ADM, USA, Lot; AE14032911) in water *ad libitum*. After 5-weeks on the HFF, a 23% increase in body weight gain was observed and deemed mice were ready for treatment intervention. Mice were then randomized into six groups: 1) normal control + vehicle (*N* = 7, Normal diet, Research diet D12450B), 2) HFF + vehicle (*N* = 7), 3) HFF + Orlistat (*N* = 7, 40 mg/kg), 4) HFF + UP601 low dose (*N* = 7, 300 mg/kg), 5) HFF + UP601 mid dose (*N* = 7, 450 mg/kg), 6) HFF + UP601 high dose (*N* = 7, 600 mg/kg) and administrations were initiated orally twice per day and sustained for 7 weeks.

The vehicle-treated animals received 0.15% xanthan gum + 0.5% Tween 80 only. The positive control, Orlistat (Lipidown Cap 120 mg, Lot #12003, Hanmi, Korea), N-Formyl-L-leucine (1S)-1-[[(2S,3S)-3-hexyl-4-oxo-2-oxetanyl]methyl]dodecyl ester, with a trade name of Xenical or Alli, is a Food and Drug Administration-approved human pancreatic lipase inhibitor, which inhibits the absorption of approximately one third of fat from ingested food that would ultimately result in weight reduction.

Water for the normal control group or fructose-enriched water for the vehicle and treatment groups was provided *ad libitum*. Animals were maintained in a temperature and air flow controlled room (22.2 °C, and 10–15 filtered air changes per hour, respectively) on a 12-h light–dark cycle with a relative humidity of 50 °C ± 10. Feed and water consumption was measured twice a week throughout treatment period. To better estimate calorie intake by mice, instead of 5 mice/cage, as of week 6, each mouse was placed in an individual cage for the whole treatment duration. All animal experiments were conducted according to institutional guidelines congruent with the guide for the care and use of laboratory animals under Institutional Animal Care and Use Committees (IACUC) Approval No.: UIK21407.

### Body composition analysis using DEXA

After 5 weeks of treatment, representative mice (2 mice from each group) were selected from the normal control, HFF, 450 mg/kg UP601 and Orlistat groups and were subjected to dual energy X-ray absorptiometry (DEXA, InAlyzer™ MEDIKORS, Korea) for body composition. The system was calibrated according to manufacturer’s instructions prior to the start of the experiment. Software integrated to the scan was used for data analysis. DEXA uses two separate low-dose X-ray exposures to read bone and soft tissue mass with a high degree of precision.

### Blood chemistry analysis for liver function and lipid profiles

At necropsy day, mice were fasted for 16 h and approximately 0.6 ~ 0.9 mL of blood sample were collected from the abdominal vein. Samples were centrifuged and serum was transferred to Biotoxtech Co., Ltd. for liver function and lipid profiles analysis. Serum levels of alanine aminotransferase (ALT), aspartate aminotransferase (AST), total cholesterol (T-Chol), triglycerides (TG), LDL- cholesterol (LDL-C), HDL- cholesterol (HDL-C) and glucose were measured using a Hitachi auto-analyzer (7180, HITACHI, Japan).

### Necropsy and tissue collection for histopathology

On the last day of the assay, all animals were exsanguinated and examined for gross pathology. Once the abdominal cavity was opened, organs were subjected to gross examination. Liver, and visceral fat pads (epididymal, retroperitoneal, peri-renal fat pad and mesenteric fat) were collected and weighed individually for organ-to-body weight ratio determination then specimens were fixed with 10% buffered neutral formalin, trimmed, processed, embedded in paraffin, sectioned, and stained with Hematoxylin & Eosin (H&E) for microscopic NASH (non-alcoholic steatohepatitis) score analysis according to the modified scoring system method of Kleiner DE et al. [29].

### Statistical analysis

All non-discrete data from clinical chemistry, body weight and food consumption were represented as mean ± S.D and were analyzed using Sigmaplot (Version 11.0). Statistical significance between groups was calculated by means of single factor analysis of variance followed by a paired t-test. *P*-values less or equal to 0.05 (*P ≤* 0.05) were considered as significant. When normality tests failed, data for non-parametric analysis were subjected to Mann–Whitney sum ranks for t-test and Kruskal-Wallis one way analysis of variance on ranks for ANOVA. Interpretations of the results were made based on findings from the in-life body weights, DEXA scan, serum biomarkers and NASH score.

## Results

### Lipolytic effects of UP601 in 3T3-L1 cells

**Fig. 1:**
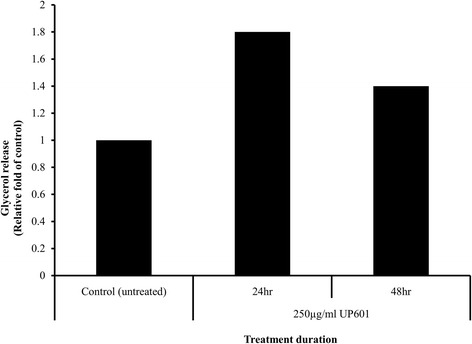
Lipolytic effect of UP601 in 3T3-L1 adipocytes. Fully differentiated adipocytes were treated with UP601 at 250 μg/ml for 24 and 48 h. Lipolysis was assessed by the amount of glycerol released into media in treated adipocytes

Up to 1.8-fold increase in the amount of glycerol released into the media was observed in adipocytes treated with UP601 for 24 h and 48 h. The lipolytic effect of UP601 was time dependent. The significant increase in glycerol release was observed after 24 h of treatment (Fig. 1).

### Effect of UP601 on body weight

**Fig. 2:**
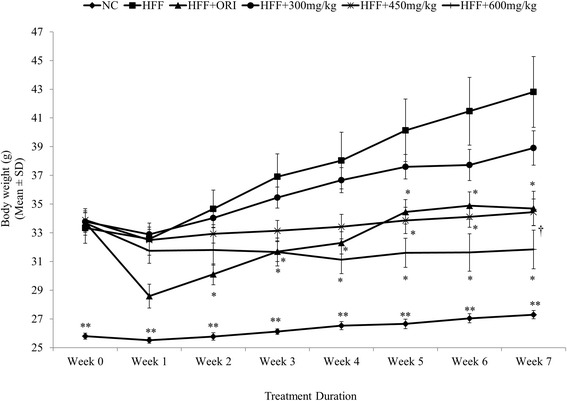
Body weight changes observed after 7 weeks of daily oral UP601 treatment in HFF fed obese C57BL/6J mice. HFF-induced obese C57BL/6J mice were treated with UP601 at oral doses of 300, 450 and 600 mg/kg for 7-weeks. Orlistat (ORI) at 40 mg/kg was used as a reference compound. *N* = 7 for each group; **P ≤ 0.0001 compared to HFF group; †*P ≤* 0.001 compared to HFF group; *P ≤ 0.05 compared to HFF group

Rapid body weight gain was observed when C57BL/6J mice were fed a 60% Kcal High-Fat-Diet and 30% fructose enriched water *ad libitum* for 5 weeks. Mice were transferred to a ‘mini-cage’ that houses a single mouse per cage for the treatment duration. After 5-weeks on the HFF, a 23% increase in body weight gain was observed and deemed mice were ready for randomization for treatment intervention and treatment was initiated. As seen in Fig. 2, a statistically significant rapid drop in body weight was observed for mice treated with 40 mg/kg/day of Orlistat for the first two weeks of the treatment period followed by a moderate body weight gain compared to vehicle treated HFF group. In contrast, mice treated with UP601 showed a dose-correlated very stable, minimal body weight gain throughout the duration of treatment. In particular, mice treated with the high dose of UP601 showed 6.4% reductions in body weight when compared to baseline. Compared to the vehicle-treated HFF group, body weight gains for mice treated with UP601 were significantly lower after 3 weeks for the 600 mg/kg/day group and after 5 weeks for the 450 mg/kg/day group, and these differences remained statistically significant for the rest of treatment duration. Statistically non-significant reductions in body weight were observed for mice treated with 300 mg/kg UP601. The percent changes in body weight at week-7 compared to week-0 (baseline) were found to be 6.4% for normal control diet, 27.3% for HFF, 2.0% for HFF + Orlistat, 14.5% for HFF + 300 mg/kg UP601, 1.3% for HFF + 450 mg/kg UP601, and −6.4% for HFF + 600 mg/kg UP601 (Fig. 2). When these changes were computed against the HFF group, it was found that mice treated with the composition UP601 showed dose correlated reductions of 9.1% for 300 mg/kg UP601, 19.6% for 450 mg/kg UP601 and 25.6% for 600 mg/kg UP601 at the end of the seven week treatment period. The positive control, Orlistat, resulted in 19.0% reduction in body weight compared to vehicle-treated HFF-fed mice (Fig. 2).

### Effect of UP601 on liver and lipid biomarkers

Abnormal lipid metabolism and excessive lipid accumulation in tissues are cardinal features of obesity as a result of consumption of high-fat in combination with high-fructose diet. The serum glucose, total cholesterol, triglyceride and LDL-cholesterol levels of mice in the HFF group were found to be 35.9, 67.2, 131.9 and 217.9% higher compared to regular diet-fed mice at the end of study, respectively (Table 1). As depicted in Table 1, the UP601 composition restored altered metabolic disturbances as demonstrated by serum liver enzymes and lipid panel levels to near normal. Mice treated with UP601 showed 9.1, 16.9 and 29.8% reductions in total cholesterol, 45.0, 55.0, 63.6% reductions in triglyceride, 34.8, 37.1 and 41.6% reductions in LDL for the 300 mg/kg, 450 mg/kg and 600 mg/kg groups, respectively, compared to vehicle-treated HFF-fed mice (Table 1). These reductions were all statistically significant compared to vehicle-treated HFF-fed mice for all the dosages except for the 300 mg/kg reduction in the total cholesterol. Improvement in dyslipidemia seemed to appear dose correlated.

**Table 1:** Liver function and lipid profiling

Group	Dose (mg/kg)	ALT (U/L)	AST (U/L)	Glucose (mg/dL)	T-chol (mg/dL)	TG (mg/dL)	LDL-C (mg/dL)	HDL-C (mg/dL)
NC	0	21.6 ± 1.7	45.3 ± 13.8	206.4 ± 46.6*	101.1 ± 17.4‡	14.1 ± 3.4†	2.8 ± 0.8‡	57.6 ± 10.4*
HFF	0	36.2 ± 21.3	58.2 ± 10.0	280.6 ± 54.6	169.4 ± 17.2	32.7 ± 11.3	8.9 ± 2.4	69.5 ± 3.4
ORI	40	17.0 ± 2.2*	45.8 ± 4.2*	238.6 ± 69.0	139.1 ± 18.0†	51.3 ± 27.1	5.0 ± 1.2†	68.6 ± 6.2
UP601	300	19.5 ± 4.8	47.7 ± 4.2*	271.3 ± 35.4	153.9 ± 9.5	18.0 ± 9.4*	5.8 ± 0.5†	65.2 ± 3.7*
UP601	450	19.8 ± 12.2	53.1 ± 19.6	219.9 ± 35.1*	140.7 ± 16.5†	14.7 ± 6.1†	5.6 ± 0.9†	65.1 ± 4.1
UP601	600	16.5 ± 7.5*	48.7 ± 19.1	186.1 ± 47.1†	118.9 ± 21.0‡	11.9 ± 4.8†	5.2 ± 1.2†	57.7 ± 10.5*

HFF-induced obese C57BL/6J mice were treated with UP601 at oral doses of 300, 450 and 600 mg/kg for 7-weeks. Orlistat (ORI) at 40 mg/kg was used as a reference compound. Serum samples were collected at necropsy. *ALT* alanine aminotransferase, *AST* aspartate aminotransferase, *T-chol* total cholesterol, *TG* triglycerides, *LDL C* low-density lipoprotein cholesterol, *HDL-C* high density lipoprotein cholesterol. *NC* Normal control + vehicle, *HFF* HFF + Vehicle, *ORI* HFF + orlistat, *UP601* HFF + indicated concentrations of UP601. *P ≤ 0.05 compared to HFF group*;* †*P ≤ 0.001* compared to HFF group*;* ‡*P ≤ 0.0001* compared to HFF group

Hyperglycemia is one of the characteristic manifestations of metabolic disorder. Treatment with UP601 reversed the abnormal glucose level near to the normal control mice fed a regular diet. Reductions of 3.2, 21.6 (*P* = 0.03) and 33.7% (*P* = 0.005) in serum glucose level were observed for mice treated with UP601 at oral doses of 300 mg/kg, 450 mg/kg and 600 mg/kg, respectively, compared to the HFF group. In contrast, mice treated with Orlistat showed a 56.9% increase in serum triglyceride, 15.0%, 17.9% (*P* = 0.01) and 43.8% (*P* = 0.005) reductions in glucose, total cholesterol and LDL, respectively, compared to vehicle treated HFF (Table 1).

Alanine aminotransferase (ALT) and aspartate aminotransferase (AST) are the two primary liver enzymes frequently used to determine liver damage. Treatment with UP601 resulted in substantial reductions in these enzymes in serum. 46.1, 45.3, and 62.7% reductions in ALT and 18.0, 8.7 and 16.3% reductions in AST levels were observed for mice treated with 300 mg/kg, 450 mg/kg and 600 mg/kg of UP601, respectively, compared to vehicle-treated HFF-fed mice (Table 1). Mice treated with Orlistat showed, 53.0 and 21.3% reductions in ALT and AST, respectively, compared to vehicle-treated HFF (Table 1).

### Effect of UP601 on body composition and relative organ weight

It has been reported that mice fed HFF to develop metabolic disorder manifested by increased abdominal fat deposition, impaired glucose tolerance, dyslipidemia, hyperinsulinemia, and increased systolic blood pressure [30, 31]. Abdominal fat deposit was effectively reduced by the mid and high dose of UP601. As seen in Table 2, the mid and high dose of UP601 efficiently decreased epididymal, retro-peritoneal, peri-renal and mesenteric fat deposit compared to vehicle-treated HFF group. It was found that mice treated with 450 mg/kg and 600 mg/kg UP601 had 23.4 and 77.3% reductions in epididymal fat, 9.5 and 75.1% reductions in retro-peritoneal fat, 44.4 and 87.8% reductions in peri-renal fat, and 50.0 and 89.1% reductions in mesenteric fat deposit, respectively, compared to vehicle treated HFF. While the effects on the epididymal, retro-peritoneal, and peri-renal fat deposits were minimal for mice treated with 300 mg/kg of UP601, a notable 27.9% reduction in the mesenteric fat deposit was observed for this group, compared to vehicle treated HFF. As expected, mice fed the HFF showed 3.96-, 3.84-, 4.13-, and 3.14-fold increase in epididymal, retro-peritoneal, peri-renal and mesenteric fat deposits, respectively, in comparison to the regular diet control mice. The epididymal, retro-peritoneal, peri-renal and mesenteric fat loss in the Orlistat-treated mice were 17.0, 16.6, 34.3 and 42.8%, respectively, compared to the vehicle-treated HFF group (Table 2).

**Table 2:** Relative organ weight for mice treated with UP601 in HFH-induced mouse obesity model

Group	Liver (%)	Fat deposit (%)				
		Epididymal	Retroperitoneal	Peri-Renal	Mesenteric	Total
NC	3.39 ± 1.47	1.75 ± 0.33‡	0.44 ± 0.14‡	0.24 ± 0.10‡	0.81 ± 0.15‡	3.22 ± 0.67‡
HFF	3.17 ± 0.47	6.93 ± 1.06	1.69 ± 0.30	0.99 ± 0.20	2.76 ± 0.97	12.36 ± 1.17
ORI (40)	3.21 ± 0.12	5.75 ± 1.59	1.41 ± 0.38	0.65 ± 0.17†	1.58 ± 0.64*	9.38 ± 2.63*
UP601 (300)	2.83 ± 0.14	6.82 ± 0.64	1.68 ± 0.14	0.82 ± 0.17	1.99 ± 0.48	11.31 ± 0.92
UP601 (450)	2.96 ± 0.16	5.31 ± 1.04*	1.53 ± 0.29	0.55 ± 0.12‡	1.38 ± 0.24	8.76 ± 1.54‡
UP601 (600)	2.99 ± 0.17	1.57 ± 1.57†	0.42 ± 0.42*	0.12 ± 0.12‡	0.30 ± 0.30†	2.41 ± 2.41‡

HFF-induced obese C57BL/6J mice were treated with UP601 at oral doses of 300, 450 and 600 mg/kg for 7-weeks. Orlistat (ORI) at 40 mg/kg was used as a reference compound. Tissue collections were carried out at necropsy. *NC* Normal control + vehicle, *HFF* HFF + Vehicle, *ORI* HFF + orlistat, *UP601* HFF + indicated concentrations of UP601 (mg/kg). * *P ≤ 0.05* compared to HFF group; † *P ≤ 0.001* compared to HFF group; ‡ *P ≤ 0.0001* compared to HFF group

**Fig. 3:**
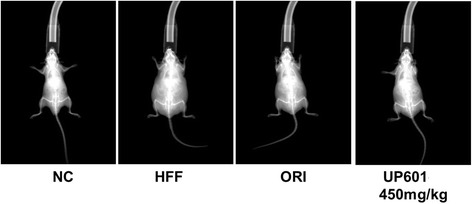
DEXA scan images for mice in the HFF-induced obesity group. HFF-induced obese C57BL/6J mice were treated with UP601 at oral doses of 300, 450 and 600 mg/kg for 7-weeks. Orlistat (ORI) at 40 mg/kg was used as a reference compound. NC: Normal control + vehicle; HFF: HFF + Vehicle; ORI: HFF + orlistat; HFF + 450 mg/kg UP601

Limited representative dual energy X-ray absorptiometry (DEXA) scan was performed to determine the lean body and fat mass distribution of sedated mice (Fig. 3). The percent body fat distribution for the HFF group was 161.5% to that of the regular diet-fed normal control group. There was no difference in the lean mass between groups. However, compared to HFF, 31.6 and 17.2% reductions in fat deposit were observed for mice treated with the mid-dose of UP601 and Orlistat, respectively. The DEXA images seemed to support the body weight data. Mice in the UP601 group appeared leaner than the orlistat or the HFF mouse.

### Effect of UP601 on liver histopathology

**Fig. 4:**
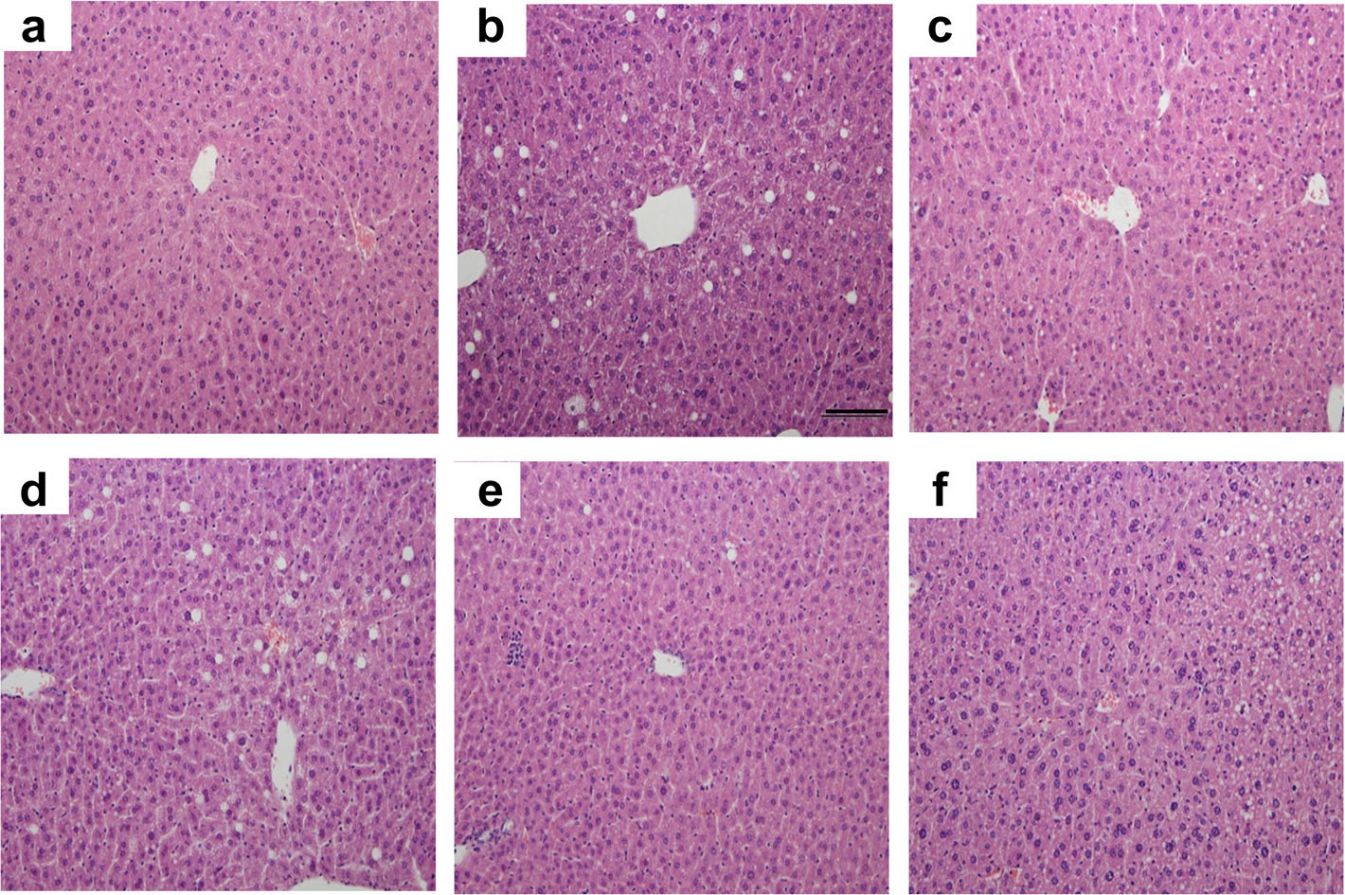
H&E staining of liver tissue. 200x magnification. **a** Normal control; **b** HFF; **c** HFF + Orlistat treated; **d** HFF + 300 mg/kg UP601; **e** HFF + 450 mg/kg UP601; **f** HFF + 600 mg/kg treated

**Fig. 5:**
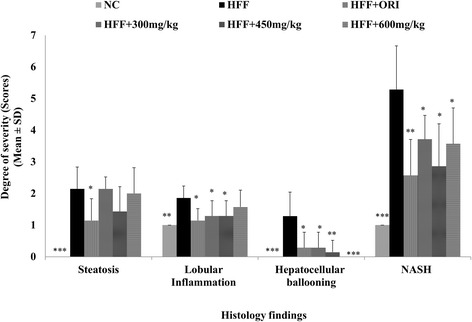
Non-alcoholic Steato-Hepatitis (NASH) Scores for mice in the HFF study group treated with Orlistat (40 mg/kg) and UP601 (300 mg/kg, 450 mg/kg and 600 mg/kg). *N* = 7 for each group, ***P ≤ 0.0001 compared to HFF group; **P ≤ 0.001 compared to HFF group;*P ≤ 0.05 compared to HFF group

Quick onsets of lipid accumulation accompanied with vacuolation are the common histopathological phenomena observed in the liver when mice are fed HFF. Histology analysis of H&E stained liver sections from the HFF groups [Fig. 4b] of mice exhibited enlargement of hepatocytes and steatosis represented by the vacuolation and microvesicular fat droplets in hepatocytes in comparison with those in the normal control mice [Fig. 4a]. Mice in the HFF group developed severe steatosis, lobular inflammation and hepatocellular ballooning. While there did not appear to be a difference in degree of steatosis between the HFF and UP601 groups [specially for the 300 mg/kg (Fig. 4d) and 600 mg/kg (Fig. 4f)], significantly improved NASH scores (*P* = 0.02, 0.01, and 0.02 for the 300 mg/kg, 450 mg/kg and 600 mg/kg, respectively) summing the effect of UP601 in steatosis, lobular inflammation and hepatocellular ballooning were observed compared to vehicle treated HFF group (Figs. 4 and 5). In particular, no hepatocellular ballooning was observed for mice treated with the high dose of UP601 (Fig. 4f). There was no obvious difference between the normal control and the mid-dose group of UP601 with regard to liver histology (Fig. 4e). Similarly, statistically significant improvement in the NASH score was also noted for the Orlistat group compared to vehicle treated HFF group (Figs. 4c and 5).

## Discussion

Excessive fat intake and consumption of soft drinks loaded with high-fructose are closely associated with increased prevalence of metabolic syndrome and may lead to non-alcoholic fatty liver disease (NAFLD) [32–34]. The past few decades’ diet westernization has resulted in significant increases in daily calorie intake because of the added fructose to easily accessible cheap food and soft drinks. For example, the typical daily consumption of fructose has escalated from 16–20 g per day (mostly from fresh fruits) to 85–100 g per day (mainly in the form of high-fructose corn syrup) within the past three decades [35]. The exposure of the liver to such a high extent of fructose could lead to rapid stimulation of lipogenesis and hepatocyte triglyceride accumulation [36]. Consumed together with fat, it renders the dietary fructose and dietary fat to serve as sources for endogenous [37] and exogenous lipids [38], and their co-presence exacerbates the prevalence of metabolic disorder. For the purpose of this simulation, the animal model was developed by feeding mice a diet containing a combination of a 60% high-fat diet with 30% fructose in water (HFF) for 5 weeks. In the current study the biochemical and biological activity of a well-defined composition, UP601, has been evaluated using this very well-established animal model of obesity that mimics human metabolic disorder exhibited as a result of consumption of the so-called western diet. For screening, a legacy mining approach was used to search Medline, pharmacopeia of traditional Chinese medicine, Ayurveda medicine, and our Phytologix collection database for plants that have potential for weight management and metabolic disorder-related indications, which led to the discovery of UP601.

Data depicted in the present study demonstrate that exposure to a combination of high-fat diet and free access to fructose in the drinking water led mice to exhibit apparent phenotypes of metabolic syndrome, including increased body weight, dyslipidemia and increased blood glucose level, confirming the role of the two high calorie dietary factors in the increased prevalence of obesity and metabolic syndrome. A 56.8% increase in body weight was observed for vehicle-treated mice fed HFF compared to vehicle-treated regular diet-fed normal control mice at the end of the study. Much of this weight gain is believed to be a result of excessive visceral fat deposit. High-fat diets and high-fructose diets have been shown to produce more rapid weight gain in rodents [39]. In contrast, these conditions were moderated by oral treatment of UP601. For example, mice treated with UP601 showed a reduced body weight gain and improved metabolic performance compared to vehicle treated HFF fed mice. These body weight reductions were analyzed and were found to be 9.1, 19.6 and 25.6% for mice treated with UP601 at oral doses of 300, 450 and 600 mg/kg, respectively, when compared to the vehicle treated HFF-fed group at week 7. Within the same treatment period, the Orlistat group had a 19.0% reduction in body weight compared to HFF. Almost complete prevention of body weight gain was observed in those groups that received higher doses (450 and 600 mg/kg) of UP601. Compared to week-0 (treatment start), the mid-dosage (450 mg/kg) of the composition maintained the initial treatment start body weight throughout the course of the treatment with only 1.3% body weight gain; the high dose (600 mg/kg) resulted in a 6.4% reduction from the initial body weight of treatment start compared to the end of treatment period. For comparison, the HFF group gained a 27.3% and the Orlistat group gained 2% of their treatment start initial body weight.

Compromised lipid metabolism and lipid accumulation in tissues are cardinal features of obesity as a result of consumption of high-fat in combination with high-fructose. As demonstrated by the liver histology and clinical chemistry data, UP601 treatment mitigated these abnormal changes. Treatments of HFF diet-induced obese mice with UP601 for 7 weeks significantly reduced total cholesterol, LDL, and triglyceride. Moreover, the hyperglycemia observed in the mice fed HFF was moderated to the level of the normal control mice fed regular rodent diet by the oral treatment of UP601. These findings are in accordance with the reported data for individual constituents of the composition. Thus, UP601 appears to possess additional beneficial effects in regulation of dyslipidemia and management of healthy blood glucose.

Liver, as a regulator of glucose and lipid metabolism, is the primary organ to be affected by continual insult from high calorie loading that could lead to fatty liver. In particular, nonalcoholic fatty liver disease exhibits a wide spectrum of indexes ranging from steatosis alone, steatosis with inflammation, steatosis with hepatocyte injury, or steatosis with sinusoidal fibrosis in relation to stages of disease progression [40, 41]. Many of these abnormal alterations were observed in vehicle-treated mice fed HFF under histopathology examinations. However, mice treated with UP601 showed significantly improved NASH scores in lobular inflammation and hepatocellular ballooning without affecting the liver enzymes (AST and ALT) suggesting the composition may have an indication in fatty liver disease without associated adverse effects. In fact, previously, *Morus* extract has been shown to reduce plasma triglycerides, hepatic fat accumulation and epididymal adipocyte size after a 12-week treatment period in HFD-induced obese mice [42]. Comparable findings were also observed for *Yerba mate* in that a decrease in the accumulation of lipids in adipocytes, and reductions in serum cholesterol, serum triglycerides, and glucose concentrations were reported when administered orally to high-fat diet-fed C57BL/6J mice daily at doses of 0.5 – 2 g/kg for 4-weeks [16].

Visceral fat deposit is one of the characteristic manifestations of obesity. In our study, mice in the UP601 group physically appeared leaner than vehicle-treated HFF-fed mice. This was demonstrated by the relative organ weight data. Mice in this group had statistically significantly reduced epididymal, retroperitoneal, peri-renal and mesenteric fat deposits, with the largest decrease in fat mass occurring in the mesenteric deposits, which was 89.1% (for 600 mg/kg UP601) less than the vehicle-treated HFF mice. This implies that the rapid increase in body weight observed in the HFF-fed mice could be as the result of visceral fat accumulation. It can also be inferred that the significant reductions in body weight noted for mice treated with UP601 could also be due to minimal visceral fat deposition. In fact, the representative data from the DEXA scan support these observations in that the reduction in body weight may be a result of low body fat accumulation.

Noteworthy anti-adipogenic effects were also observed for the composition in vitro. UP601 stimulates lipolysis in 3T3-L1 adipocytes. This finding is consistent with the reduced preliminary body fat distribution data from the DEXA scan and relative organ weights for visceral fat. Previously it has been reported for *Yerba mate* to inhibit adipogenesis [43] and triglyceride accumulation in 3T3-L1 adipocytes [44]. Extracts from another component of the composition, *Magnolia,* have also effectively reduced lipid accumulation in 3T3-L1 adipocytes [45]. Moreover, extracts from *M. alba* have also shown inhibitory effects in the proliferation and differentiation of preadipocytes [46] and the accumulation of triglyceride in 3T3-L1 cells [47]. Thus, the anti-obesity effect observed in the current study could partially be explained by the lipolytic activity of UP601.

Furthermore, augmented studies have been documented in relation to *Morus*, *Magnolia* and *Yerba mate* plant extracts that contain bioactive compounds possessing metabolic disease-modifying activities beneficial to the effects observed in the present study. For example, extracts from *Magnolia* have shown decreases in fasting blood glucose and plasma insulin in type 2 diabetic rats [48], amelioration of body fat accumulation, insulin resistance, and adipose inflammation in high-fat fed mice [49], stimulation of glucose uptake in insulin-sensitive and insulin-resistant murine and human adipocytes using the insulin signaling pathway [50], and control of elevated stress-related cortisol level [51] in human. The beneficial moderation in body weight and metabolic markers observed in the current study could partially be explained by the inherent activities of *Magnolia* extract contributing to the composition as described in this summary.

Likewise, significant studies have also demonstrated that *Yerba mate* extracts alleviates weight gain and improves plasma glucose and lipid profiles in animals [52]. It has been reported that HFD-induced obese mice treated orally with *Yerba mate* extract for 8 weeks at a dose of 1 g/kg displayed a marked attenuation of weight gain and adiposity, a decrease in epididymal fat pad weight, and restoration of the serum levels of cholesterol, triglycerides, LDL cholesterol, and glucose. These results were identical to what were observed in our study. Additionally, significantly lowered body weight, visceral fat-pad weights, blood and hepatic lipid, glucose, insulin, and leptin levels were observed in HFD rats treated with a *Yerba mate* extract formulated diet for 60-days [53]. Here, again, the composition UP601 might be assumed to adapt the suggested mechanism of action and activities of *Yerba mate* as shown by data depicted in the current study.

The impact of *Morus* extracts in metabolic disorders like diabetes and obesity have also been reported. Significantly low body weight gain [42], improved hyperglycemia and associated complications in the diabetic rats [54], decreased body weight, adiposity, and hepatic lipid accumulation in diet-induced obese mice [55], decreased expression of white muscle adipocytokines in db/db mice [56] and blood glucose level in alloxan diabetic mice [57] are some of the beneficial effects documented for this plant. Previously, it has also been reported that *Morus* extract increases adiponectin level in adipocytes [58], increases glucose uptake and GLUT4 translocation in adipocytes [58], and inhibits α-glucosidase [59] and intestinal disaccharidase [60] activity. In support of the other two components of the composition, *Morus* extract also reinforces the potential of UP601 in weight management as a result of the collective mechanisms of action contributed by all the three components. Therefore, the data depicted in the current study ensures the merit of combining these plant extracts for a better outcome targeting multiple pathways that are indicated in various aspects of metabolic disorders.

The current study was not without a limitation. While the combination of fructose from drinking water and high-fat diet was assumed to cause accelerated body weight gain, the actual calorie intake from food and the drinking water per day per animal could not be accurately determined due to the spillage of water from the Lixit. As a result, the proportion of weight gain to the change in calorie intake due to treatment could not be confirmed. Another limitation for this study was the number of animals per group selected for DEXA scan. Only two animals per group were analyzed for body composition. The 300 mg/kg and 600 mg/kg UP601 were excluded from the body composition analysis. Hence, meaningful quantitative fat and lean mass data to support the relative organ weight findings could not be generated.

Although the multiple mechanisms by which UP601 may impact lipid and carbohydrate metabolism are plausible according to its individual components in vitro and in vivo studies from the literature, further additional studies are needed to determine the possible thermogenic (contribution from *Yerba mate*), lipolytic (contribution from all three components) and anorectic (contribution from *Morus* and *Magnolia*) effects of the composition.

## Conclusion

To sum up, we have demonstrated the anti-obesity activity of a standardized composition UP601 from the historically well-known plants: *Morus alba*, *Yerba mate* and *Magnolia officinalis*. Oral administration of UP601 to high-fat and high-fructose-fed mice for 7 weeks resulted in a significant reduction in body weight gain. The composition also moderated dyslipidemia and hyperglycemia in vivo and acted as a lipolytic in vitro. While pre-clinical to clinical data translation requires human clinical trial validation and safety study, the data depicted here in conjunction with the individual components’ safe historical usage suggest UP601 could potentially be considered as a natural alternative for metabolism management and maintaining a healthy body weight.

## Abbreviations

ALT: Alanine transaminase
ANOVA: Analysis of variance
AST: Aspartate aminotransferase
DEXA: Dual energy X-ray absorptiometry
DMEM: Dulbecco’s modified eagle’s medium
FBS: Fetal bovine serum
GLUT4: Glucose transporter type 4
H&E: Hematoxylin & Eosin
HDL-C: High-density lipoprotein-cholesterol
HFD: High-fat diet
HFF: High-fat high-fructose
IACUC: Institutional Animal Care and Use Committees
IBMX: 3-isobutyl-1-methylxanthine
LDL-C: Low-density lipoprotein-cholesterol
NAFLD: Non-alcoholic fatty liver disease
NASH: Non-alcoholic steatohepatitis
ORI: Orlistat
T-Chol: Total cholesterol
TG: Triglycerides
UP601: Standardized blend of extracts from *Morus alba, Yerba mate and Magnolia officinalis*
